# Proteasome inhibitors modulate anticancer and anti-proliferative properties via NF-kB signaling, and ubiquitin-proteasome pathways in cancer cell lines of different organs

**DOI:** 10.1186/s12944-018-0697-5

**Published:** 2018-04-02

**Authors:** Asaf A. Qureshi, Eleanor G. Zuvanich, Dilshad A. Khan, Shahida Mushtaq, Neerupma Silswal, Nilofer Qureshi

**Affiliations:** 1 Department of Basic Medical Science, School of Medicine, University of Missouri-Kansas City, 2411 Holmes Street, Kansas City, MO 64108 USA; 2 Department of Chemical Pathology and Endocrinology, Armed Forces Institute of Pathology and National University of Medical Science, Rawalpindi, 64000 Pakistan; 3 Pharmacology and Toxicology, School of Pharmacy, University of Missouri-Kansas City, 2464 Charlotte Street, Kansas City, MO 64108 USA

**Keywords:** Potent anticancer compounds, Several cancer cell lines (Hela, Liver, Pancreas, Prostate, Breast, Lung, Melanoma, B-lymphocytes, T-cells, Inflammatory biomarkers

## Abstract

**Background:**

Cancer is second most common cause of death in the United State. There are over 100 different types of cancer associated with different human organs, predominantly breast, liver, pancreas, prostate, colon, rectum, lung, and stomach. We have recently reported properties of pro-inflammatory (for treatment of various types of cancers), and anti-inflammatory (for cardiovascular disease and diabetes) compounds. The major problem associated with development of anticancer drugs is their lack of solubility in aqueous solutions and severe side effects in cancer patients. Therefore, the present study was carried out to check anticancer properties of selected compounds, mostly aqueous soluble, in cancer cell lines from different organs.

**Methods:**

The anticancer properties, anti-proliferative, and pro-apoptotic activity of novel naturally occurring or FDA approved, nontoxic, proteasome inhibitors/activators were compared. In addition to that, effect of δ-tocotrienol on expression of proteasome subunits (X, Y, Z, LMP7, LMP2, LMP10), ICAM-1, VCAM-1, and TNF-α using total RNAs derived from plasmas of hepatitis C patients was investigated.

**Results:**

Our data demonstrated that following compounds are very effective in inducing apoptosis of cancer cells: Thiostrepton, dexamethasone, 2-methoxyestradiol, δ-tocotrienol, quercetin, amiloride, and quinine sulfate have significant anti-proliferation properties in Hela cells (44% - 87%) with doses of 2.5–20 μM, compared to respective controls. Anti-proliferation properties of thiostrepton, 2-methoxyestradiol, δ-tocotrienol, and quercetin were 70% - 92%. However, thiostrepton, dexamethasone, 2-methoxyestradiol, δ-tocotrienol, quercetin, and quinine sulphate were effective in pancreatic, prostate, breast, lungs, melanoma, Β-lymphocytes, and T-cells (Jurkat: 40% to 95%) compared to respective controls. In lung cancer cells, these compounds were effective between 5 and 40 μM. The IC_50_ values of anti-proliferation properties of thiostrepton in most of these cell lines were between doses of 2.5–5 μM, dexamethasone 2.5–20 μM, 2-methoxyestradiol 2.5–10 μM, δ-tocotrienol 2.5–20 μM, quercetin 10–40 μM, and (−) Corey lactone 40–80 μM. In hepatitis C patients, δ-tocotrienol treatment resulted in significant decrease in the expression of pro-inflammatory cytokines.

**Conclusions:**

These data demonstrate effectiveness of several natural-occurring compounds with anti-proliferative properties against cancer cells of several organs of humans. Thiostrepton, dexamethasone, 2-methoxyestradiol, δ-tocotrienol and quercetin are very effective for apoptosis of cancer cells in liver, pancreas, prostate, breast, lung, melanoma, Β-lymphocytes and T-cells. The results have provided an opportunity to test these compounds either individually or in combination as dietary supplements in humans for treatment of various types of cancers.

## Background

Cancer is second most common cause of death in the United States. There are over 100 different types of cancer associated with different organs of human, and more than 575,000 people die of various types of cancer, predominantly breast, liver, pancreas, prostate, colon, rectum, lung, and stomach. Therefore, cancer is considered one of the leading causes of morbidity and mortality worldwide. The increase in cancer rates in these organs is due to different types of lifestyles in the world. The four major factors of lifestyle related to certain cancers are an unhealthy diet, tobacco use, lack of exercise and excessive exposure to ultraviolet radiation [1]. These factors also influence genes and molecular processes that result in the malignant transformation of human cells [1]. Therefore, cancer can be due to genetic predisposition inherited from family member, and it is possible one born with certain genetic mutations or a fault in a gene will be more likely to develop cancer later in life [2]. Moreover, a number of possible cancer-causing mutations occurring in our DNA during aging, thus age also make an important risk factor for cancer. Several viruses have also involved in developing cancer in humans, such as hepatitis B and C [2]. There is no doubt that an early detection of cancer can greatly improve the odds of successful treatment and survival. The consumption of healthy diet (low in fat and rich in fresh fruits, vegetables, whole grains) is as a deterrent of several types of cancer [2].

The most common cause of mortality in the United States is cancer, as a disease requires urgent improvement of therapeutic strategies. Thiostrepton, a broad-spectrum antibiotic that inhibits cell growth in a variety of human cancer cell lines [3–5]. The apoptotic activity of thiostrepton is via proteasome inhibition, resulting in the stabilization of certain proteins proves fatal to cancer cells [6]. In this regard, a protein “forkhead box M1 transcription factor” (FOXM1) over-expressed in a variety of human cancers [7], and inhibition of FOXM1 by thiostrepton may contribute to the anticancer activity [8]. The major issues associated with current anticancer drugs treatment are their insolubilities in aqueous solutions and severe side effects in cancer patients. The clinical application of most of the known proteasome inhibitors as chemotherapeutic agents has also been associated with high toxicity levels, which can be overcome by using a nanoparticle-protected delivery system [9, 10]. In the case of other hydrophobic drugs was their insolubility in aqueous solutions (medium). Therefore, the present study was carried out to investigate anticancer properties of selected compounds, (mostly aqueous-soluble) on several cancer cell lines derived from different organs.

The present investigation was initiated to find anti-proliferative, and pro-apoptotic activity of novel naturally-occurring or commercially available FDA approved nontoxic proteasome inhibitors/activators containing β-lactone moiety in their molecule as is found in proteasome inhibitor, lactacystin [11, 12]. In our earlier studies, we reported that proteasome is a pivotal regulator of inflammation, which modulates the several inflammatory biomarkers in response to a variety of stimuli. We and others reported that cell death occurs because of blocking proteolytic activity of proteasome mediated by the 20S proteasome. Proteasome is a hollow complex of three proteolytic subunits, X, Y, Z, with chymotrypsin-like, post-acidic, and trypsin-like activities, respectively [11]. A variety of inflammatory stimuli induce alteration in newly assembled “immunoproteasomes” in which X, Y, and Z subunits are partially replaced by LMP7, LMP2 and LMP10, respectively [11]. We have also demonstrated that some of these compounds impact proteasome activities depending on its 6 protease subunit sites (X,Y, Z, LMP7, LMP2 and LMP10) and signaling pathways (inhibition of NF-κB, cleavage of p-IκB) leading to LPS-induced production of nitric oxide and secretion of TNF-α in murine macrophages [11]. Moreover, we have demonstrated that tocotrienols were able to inhibit or activate proteasome’s activities depending on their concentrations in RAW 264.7, and in LPS-induced thioglycolate-elicited peritoneal macrophages prepared from C57BL/6, BALB/c, and PPAR-α knockout mice [11].

Recently, we identified two sets of compounds, which act as pro-inflammatory and anti-inflammatory in different experimental conditions [13]. The first set consisted of (−) Corey lactone, ouabain, ampicillin, ascorbic acid, codeine, amiloride-HCL, and second set consisted of thiostrepton, rifampicin, dexamethasone, δ-tocotrienol, 2-hydroxyestradiol, 2-methoxyestradiol, 25-hydroxycholesterol, nicotinic acid, vitamin D_3_, and resveratrol are potent inhibitors of chymotrypsin-like activity of 20S rabbit muscle proteasomes. The effects of these compounds are concentration-dependent [11–13]. These two sets also have similar effects on the levels of secretion of TNF-α tested in vitro in LPS-induced thioglycolate-elicited peritoneal macrophages of PPAR-α knockout mice [13]. However, these compounds also blocked production of nitric oxide due to lack of activation of NF-κB, and degradation of p-IκB. Moreover, these compounds up-regulate/down-regulate gene expression and secretion of TNF-α and down-regulated production of nitric oxide, IL-1β, IL-6 and iNOS tested in LPS-induced peritoneal macrophages of PPAR-α knockout mice that exhibit strong activation of inflammatory cytokines in response to agonist [13].

**Fig. 1:**
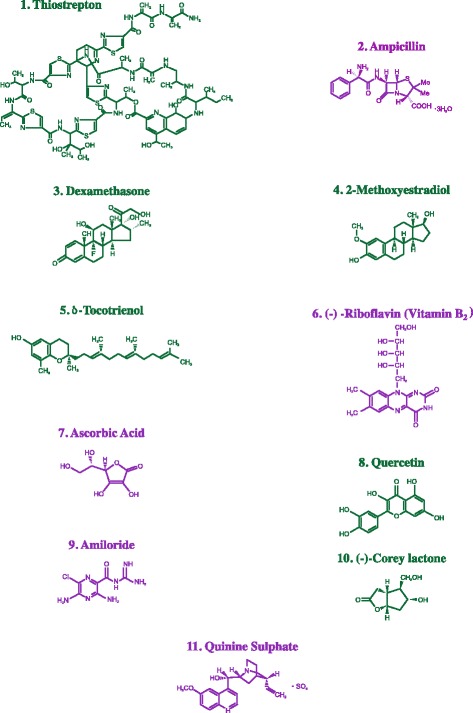
Chemical structures of dietary nutritional supplements used in the study

These results prompted us for the first time to evaluate the dose-dependent anticancer effects of these anti-inflammatory compounds (thiostrepton, ampicillin, dexamethasone, 2-methoxyestradiol, δ-tocotrienol, (−) riboflavin, ascorbic acid, quercetin, amiloride-HCL, (−) Corey lactone, and quinine sulphate) in Hela, liver, pancreatic, prostate, breast, lung, melanoma, Β-lymphocyte, and T-cells (Jurkat) cancer cell lines. Most of these compounds have the β-lactone moiety in their molecular structures (Fig. 1). Resveratrol was not included in present investigation since several investigators have already reported its effectiveness in different types of cancer cells [13, 14]. Furthermore, its multiple properties against tumorigenesis as anti-xenobiotic properties (anti-initiation) from its more general cellular effects as anti-promotion and anti-progression is adequately explained in this review article [15]. Since tocotrienols were effective in vitro and in vivo as mentioned earlier, higher doses of δ-tocotrienol acts as pro-inflammatory agents [13]. A preliminary pilot study was carried out on the effect of δ-tocotrienol feeding in hepatitis C patients on six protease subunits (X, Y, Z, LPM7, LMP2 and LMP10), ICAM-1, VCAM-1, and TNF-α using total blood mRNA of these patients.

## Methods

### Materials

Dulbecco’s Modified Eagle Medium (DMEM), heat-inactivated low-endotoxin fetal bovine serum (FBS), and gentamicin purchased from Cambrex (Walkersville MD, USA) for tissue culture. All other cancer cell lines were purchased from American Type Culture Collection (Manassas, VA, USA). Most of these following compounds (thiostrepton, ampicillin, dexamethasone, 2-methoxyestradiol, (−) riboflavin, ascorbic acid, amiloride-HCL, (−) Corey lactone, and quinine sulphate) were purchased from Sigma-Aldrich Chemical Co. (St. Louis, MO, USA), and quercetin was purchased from Alfa Aesar (Johnson Matthey Co. Lancastor, UK). The 50% purified δ-tocotrienol fraction from annatto seeds was purchased from American River (Boston, MA, USA). RNeasy mini kit was purchased from QIAGEN Sciences (Germantown, MD, USA). DeltaGold (125 mg soft gels) from annatto seeds (composition 90% δ-tocotrienol + 10% γ-tocotrienol) was obtained as a gift by American River Nutrition, Inc. (Hadley, MA. USA). The hepatitis C antibody test kit was purchased from Sigma Chemical Co., St. Louis, USA. The second kit for diagnosing hepatitis C test is RNA (PCR) test. Pure total mRNA was obtained from the EDTA treated fresh whole blood using total RNA purification kit # 17200 (NORGEN Bioteck Corporation, Thorold, ON, Canada).

### Purification of δ-tocotrienol from 50% purified fraction of annatto seeds

The δ-tocotrienol was purified from 50% purified fraction of annatto seeds, as described previously [16]. The purity of δ-tocotrienol was established by high-pressure liquid chromatography (HPLC), against its standard as reported earlier [16].

### Cell culture and maintenance

The different cancer cells of various human organs origin (liver, pancreatic, prostate, breast, lung, melanoma, Β-lymphocytes and T-cells (Jurkat) were maintained in DMEM medium supplemented with 10% heat inactivated FBS and 10 mg/mL, gentamicin at 37 °C in a humidified atmosphere with 5% carbon dioxide (CO_2)_ and 95% oxygen (O_2),_ as described previously [17]. The experimental procedures involving various human organs of different cancer cell lines reviewed and approved by the Institutional Animal Care and Use Committee, School of Medicine, University of Missouri, Kansas City, MO.

Cancer cell lines (1 × 10^5^) from various organs were seeded in 48 well tissue culture plates with 900 μl of medium containing 0.2% dimethyl sulfoxide of different type of cancer cell lines (Hela cell, liver, pancreas, prostate, breast, lung, melanoma, Β-lymphocytes, T-cells [Jurkat]), were incubated at 37 °C for 2 h. After 2 h, different concentrations (100 μl of 2.5, 5, 10, 20, 40, or 80 μM) of each compound was added to each well in triplicate, and incubated for 48 h at 37 °C in a humidified atmosphere of 5% CO_2_. This was followed by counting the number of living cells of each well using MTT method [18, 19].

### Detection of cell viability

Viability of cancer cells after treatment with different concentrations of each compound was determined by trypan-blue dye exclusion method or by a quantitative colorimetric assay with 3-(4, 5)-dimethyl-thiozol-2, 5-diphenyltetrazolium bromide (MTT) as reported earlier [18, 19].

### Impact of δ-tocotrienol in hepatitis C patients

This study was carried out in the Pakistan Ordinance Factory (POF) Hospital, Wah Cantonment, Rawalpindi, 64,000, Pakistan, in collaboration with the Department of Basic Medical Sciences, University of Missouri-Kansas City, MO, USA. The study protocol registered (IRB # 129–2015) was approved by Institutional Review Board of POF, Rawalpindi, Pakistan. The study was carried out under a FDA approved IND number 36906**.**

### Study design

Personal characteristics including demographic and disease history was obtained from the subjects. Anthropometric parameters including body weight and height measured using standard protocols, Systolic and diastolic blood pressures were measured on the right upper arm using an electronic blood pressure monitor. Body mass index (BMI) calculated as weight (kg/m^2^) divided by height (cm). All participants were asked to maintain their usual lifestyle.

#### The inclusion criteria

Adults male/female, age > 25 years with hepatitis C infection (using Anti-HCV Test of blood, or RNA = PCR test) were included in the study.

#### The exclusion criteria

Any subject having weight (> 125% of Metropolitan Life relative weights), taking anti-inflammatory drugs in the last 2 weeks were excluded. The subjects with elevated serum transaminase activity, serum urea, glucose, thyroid stimulating hormone (TSH), liver, renal, diabetes, and thyroid diseases were excluded from this study. A total of 14 (*n* = 14) hepatitis C patients were enrolled in this study, out of these total RNAs of 5 patients randomly selected were combined, purified and concentrated into two fractions of 10 μL of each (first wash and second wash from columns) to carry out further studies.

### Experimental design

#### Analyses of total RNAs from plasma after feeding δ-tocotrienol for 6-weeks to hepatitis C patients

The participants were administered four capsules of 125 mg (500 mg/d; two at 8 am and two at 8 pm after breakfast and dinner, respectively) for 6-weeks. At the end of the treatment, blood samples in plan and EDTA coated tubes collected of each participant to purify total RNAs for the estimation of several inflammatory biomarkers. Serum Bilirubin, ALT and creatinine were measured by standard clinical laboratory methods (ADVIA® 1800: Siemens Healthcare Diagnostics, New York, USA).

The pure total RNA was extracted from plasma of EDTA treated fresh whole blood of each subject fed δ-tocotrienol (500 mg/d) for 6 weeks, using total RNA purification kit # 17200 (NORGEN Bioteck Corporation, Thorold, ON, Canada). The purity of total RNA carried out by measuring the absorption at several wavelengths using a Thermo Scientific NanoDrop 1000 Spectrophotometer. The purity of total RNA was determined by the ratio of 260/280 (2.02–2.08).

#### Expression of important inflammatory biomarkers and proteasome subunits of total RNAs by real time (RT-PCR) of plasma obtained after feeding δ-tocotrienol (500 mg/d) for 6-weeks to hepatitis C patients

Five randomized samples of total RNAs of hepatitis C patients were selected (pre-dose and post-dose) and combined separately. Each combined samples of total RNAs were purified by using Biostic Blood Total RNA Isolation Kit (MOBIO Laboratories, Inc.). The purified total RNAs were further purified and concentrated to 10.0 μl by using by Gene Jet RNA Clean up and Concentration Micro Kit (Thermo Scientific, EU, Lithuania). The key cytokines and other biomarkers were estimated by real-time RT-PCR, which was carried out on total RNA isolated from plasma samples of hepatitis C patients (pre-dose and post-dose). Taqman primer-probes and Step-one RT-PCR kit (Life Technologies (Foster City, California) were used for these estimations. All reactions performed in triplicate using equal amount of total RNA per reaction. Reverse-transcriptase step involved incubation at 48 °C for 15 min. The real-time (RT-PCR) cycling conditions included an initial denaturation at 95 °C for 10 min, followed by 40 cycles of 95 °C for 15 s, and 60 °C for 60 s. Real-time PCR assays were completed using a Step-one plus Real time-PCR kit. Relative gene expression of hepatitis C patients of pre-dose versus post-dose samples were normalized (2^−ΔΔC^_T_ analysis) to GAPDH*.*

### Statistical analyses

The data were analyzed by using the GLM procedure of SAS (Statistical Analysis System; version 4.01, Abacus Concepts, Berkeley, CA) for personal computers to test the study hypothesis. Data reported as mean ± SD (Standard Deviation). Stat View software (version 4.01, Abacus Concepts, Berkeley, CA) used for the analyses of treatment-mediated effects as compared to control group. Treatment-mediated differences detected with a two-way ANOVA, and when the F test indicated a significant effect, differences between the means analyzed by a Fisher protected least significant difference test. Data reported as means ± SD/SE in text and Tables. The statistical significance level was set at 5% (*P* < 0.05).

## Results

### Dose-dependent response of anticancer properties of various compounds in cancer cells (Hela, liver and pancreas)

The anti-cancer properties of thiostrepton (10 μM; 24%), dexamethasone (40 μM; 51%), 2-methoxyestradiol (25 μM; 48%), δ-tocotrienol (5 μM; 45%), quercetin, amiloride, and quinine sulphate (10 μM; 51%, 51%, 27%), respectively were observed in Hela cells compared to respective controls (Table 1). Ampicillin, (−) riboflavin, ascorbic acid, Amiloride-HCL, and (−) Corey lactone required higher doses of 160–320 μM to achieve 50% cell death in Hela cells (Table 1).

**Table 1:** Dose response of anticancer properties of various compounds in Hela cells

Concetrations	Thiostrepton	Ampicillin	Dexamethasone	2-Methoxyestradiol	δ-Tocotrienol	(−) Riboflavin	Ascorbic acid	Quercetin	Amiloride-HCL	(−)Corey lactone	Quinine Sulphate
in μM	1	2	3	4	5	6	7	8	9	10	11
MC^a^ 0.0	43.0	46.0	33.0	33.0	46.0	43.0	43.0	45.0	55.0	55.0	45.0
MC + DMSO^b^ 0.0	54.0 (100)^c^	43.0 (100)	34.0 (100)	34.0 (100)	41.0 (100)	42.0 (100)	40.0 (100)	56.0 (100)	54.0 (100)	54.0 (100)	55.0 (100)
2.5	51.0 (94)	41.0 (95)	26.0 (76)	21.0 (62)	26.0 (63)	40.0 (95)	33.0 (83)	46.0 (82)	41.0 (760)	49.0 (91)	52.0 (93)
5.0	36.0 (67)	38.0 (88)	22.0 (65)	**16.0 (47)**	24.0 (59)	40.0 (95)	32.0 (80)	45.0 (80)	37.0 (69)	45.0 (83)	40.0 (71)
10.0	**13.0 (24)**	36.0 (84)	23.0 (68)	12.0 (35)	**13.0 (32)**	34.0 (81)	27.0 (68)	42.0 (75)	**28.0 (52)**	47.0 (87)	**24.0 (44)**
20.0	11.0 (20)	33.0 (77)	20.0 (59)	9.0 (25)	11.0 (27)	32.0 (76)	28.0 (70)	**27.0 (48)**	31.0 (57)	45.0 (83)	15.0 (27)
40.0	9.0 (16)	30.0 (70)	22.0 (68)	7.0 (21)	9.0 (22)	29.0 (69)	24.0 (60)	23.0 (41)	35.0 (65)	42.0 (78)	11.0 (20)
80.0	0.0 (0)	32.0 (74)	22.0 (68)	3.0 (9)	10.0 (24)	27.0 (64)	**22.0 (52)**	20.0 (36)	31.0 (57)	36.0 (67)	4.0 (7)

^a^Medium + Cells; ^b^Medium + Cells + 0.2%DMSO (dimethyl sulphoxide); ^c^Percentage of control values are in parentheses

Boldfaced values signify the most effective doses

Similarly, anticancer properties of thiostrepton (2.5 μM; 53%), 2-methoxyestradiol (5 μM; 45%), δ-tocotrienol (20 μM; 53%), quercetin (20 μM; 32%) were observed for liver cancer cells apoptosis (Table 2). Ampicillin, dexamethasone, (−) riboflavin, ascorbic acid kills cells at much higher doses (> 320 μM) for their anticancer properties (Table 2). Whereas, amiloride-HCL, (−) Corey lactone, and quinine sulphate (80 μM; 53% - 58%) required 160 μM to pre-dose more than 50% cancer cells apoptosis of liver (Table 2). Thiostrepton (10 μM; 16%), 2-methoxyestradiol (40 μM; 42%), δ-tocotrienol (10 μM; 36%), and quercetin (40 μM; 45%) induced more than 50% apoptosis of cancerous human pancreatic cells (Table 3). Whereas, remaining seven compounds required doses of > 320 μM to produce 50% apoptosis of pancreatic cancer cell line (Table 3).

**Table 2:** Dose-response of anticancer properties of various compounds in liver cancer cells

Concentrations	Thiostrepton	Ampicillin	Dexamethasone	2-Methoxyestradiol	δ−Tocotrienol	(−) Riboflavin	Ascorbic Acid	Quercetin	Amiloride-HCL	(−) Corey Lactone	Quinine Sulphate
in μM	1	2	3	4	5	6	7	8	9	10	11
MC^a^ 0.0	63.0	69	69.0	69.0	69	73	54	73	54	54	83.0
MC + DMSO^b^ 0.0	80.0 (100)^c^	80.0 (100)	73.0 (100)	73.0 (100)	73.0 (100)	80.0 (100)	56.0 (100)	80.0 (100)	56.0 (100)	56.0 (100)	80.0 (100)^c^
2.5	42.0 (53)	77.0 (96)	71.0 (97)	48.0 (71)	70.0 (96)	73.0 (91)	50.0 (89)	66.0 (83)	55.0 (98)	60.0 (107)	71.0 (89)
5.0	**13.0 (16)**	68.0 (85)	68.0 (93)	**33.0 (48)**	57.0 (78)	75.0 (94)	48.0 (86)	59.0 (74)	49.0 (88)	58.0 (104)	68.0 (85)
10.0	7.0 (8)	74.0 (93)	66.0 (90)	27.0 (39)	39.0 (53)	71.0 (89)	49.0 (88)	52.0 (65)	50.0 (89)	49.0 (88)	66.0 (83)
20.0	1.0 (1)	72.0 (90)	64.0 (88)	20.0 (20)	**35.0 (48)**	73.0 (91)	45.0 (80)	**26.0 (33)**	48.0 (86)	51.0 (91)	68.0 (85)
40.0	2.0 (3)	71.0 (89)	60.0 (82)	18.0 (26)	26.0 (36)	70.0 (86)	49.0 (88)	23.0 (29)	51.0 (91)	49.0 (88)	63.0 (79)
80.0	0.0 (0)	72.0 (90)	60.0 (82)	11.0 (16)	5.0 (7)	66.0 (83)	42.0 (75)	14.0 (18)	46.0 (82)	42.0 (75)	48.0 (60)

^a^Medium + Cells; ^b^Medium + Cells + 0.2% DMSO (dimethyl sulphoxide); ^c^Percentage of control values are in parentheses

Boldfaced values signify the most effective doses

**Table 3:** Dose-respnse of anticancer properties of various compounds in human pancreas cancer cells

Concentrations	Thiostrepton	Ampicillin	Dexamethasone	2-Methoxyestradiol	δ-Tocotrienol	(−) Riboflavin	Ascorbic Acid	Quercetin	Amiloride-HCL	(−) Corey Lactone	Quinine Sulphate
in μΜ	1	2	3	4	5	6	7	8	9	10	11
MC^a^ 0.0	47.0	47.0	47.0	47.0	55	55	44	40	53	40	44.0
MC + DMSO^b^ 0.0	48.0 (100)^c^	48.0 (100)	48.0 (100)^c^	48.0 (100)^c^	59.0 (100)	59.0 (100)	46.0 (100)	44.0 (100)	49.0 (100)	45.0 (100)	45.0 (100)^c^
2.5	43.0 (71)	45.0 (91)	44.0 (92)	41.0 (85)	47.0 (80)	55.0 (93)	42.0 (91)	40.0 (91)	46.0 (94)	44.0 (98)	41.0 (91)
5.0	**14.0 (29)**	42.0 (88)	46.0 (96)	38.0 (79)	36.0 (61)	48.0 (81)	40.0 (87)	32.0 (73)	45.0 (92)	41.0 (91)	38.0 (84)
10.0	8.0 (17)	40.0 (83)	44.0 (92)	32.0 (67)	**29.0 (49)**	46.0 (80)	42.0 (91)	**16.0 (36)**	43.0 (88)	39.0 (87)	36.0 (80)
20.0	2.0 (4)	38.0 (79)	42.0 (88)	30.0 (63)	18.0 (31)	48.0 (81)	38.0 (83)	12.0 (27)	39.0 (80)	44.0 (98)	38.0 (84)
40.0	1.0 (2)	40.0 (83)	42.0 (88)	**20.0 (42)**	6.0 (10)	43.0 (73)	39 (85)	10.0 (23)	40.0 (82)	40.0 (89)	40.0 (89)
80.0	1.0 (2)	40.0 (83)	42.0 (88)	15.0 (31)	2.0 (3)	45.0 (76)	38.0 (83)	8.0 (18))	40.0 (82)	42.0 (93)	40.0 (89)

^a^Medium + Cells; ^b^Medium + Cells + 0.2% DMSO (dimethyl sulphoxide); ^c^Percentage of control values are in parentheses

Boldfaced values signify the most effective doses

### Dose-dependent response of anticancer properties of various compounds in cancer cells obtained from prostate, breast, and lung

The anticancer properties of thiostrepton, ampicillin, dexamethasone, 2.methoxyestradiol, δ-tocotrienol, quercetin, (−) Corey lactone and quinine sulphate resulted in > 50% cell apoptosis with very small doses of 2.5 μM - 10 μM, compared to respective controls in prostate cancer cells (Table 4). However, thiostrepton (5 μM; 35%), 2-methoxyestradiol (5 μM; 49%), δ-tocotrienol (80 μM; 51%), quercetin (40 μM; 52%), and amiloride-HCL (40 μM; 52%) were more effective for apoptosis of breast cancer cells, compared to respective controls (Table 5).

**Table 4:** Dose-response of anticancer properties of various compounds in prostate cancer cells

Concentrations	Thiostrepton	Ampicillin	Dexamethasone	2-Methoxyestradiol	δ-Tocotrienol	(−) Riboflavin	Ascorbic Acid	Quercetin	Amiloride-HCL	(−) Corey Lactone	Quinine Sulphate
in μΜ	1	2	3	4	5	6	7	8	9	10	11
MC^a^ 0.0	32.0	32	45.0	46.0	31	32	38	38	36	36	38.0
MC + DMSO^b^ 0.0	37.0 (100)^c^	37.0 (100)^c^	52.0 (100)^c^	52.0 (100)^c^	36.0 (100)	36.0 (100)	43.0 (100)	43.0 (100)	39.0 (100)	39.0 (100)	43.0 (100)^c^
2.5	**20.0 (54)**	24.0 (65)	41.0 (79)	40.0 (77)	28.0 (78)	37.0 (103)	38.0 (88)	40.0 (95)	38.0 (97)	39.0 (100)	36.0 (84)
5.0	7.0 (10)	**18.0 (49)**	28.0 (88)	38.0 (73)	23.0 (64)	35.0 (97)	39.0 (91)	35.0 (81)	36.0 (92)	40.0 (103)	33.0 (77)
10.0	6.0 (16)	16.0 (43)	**26.0 (50)**	**16.0 (31)**	**17.0 (47)**	32.0 (89)	32.0 (74)	32.0 (74)	37.0 (95)	33.0 (85)	31.0 (72)
20.0	2.0 (5)	13.0 (35)	28.0 (54)	13.0 (25)	20.0 (56)	27.0 (75)	27.0 (63)	**19.0 (44)**	40.0 (103)	36.0 (92)	**17.0 (40)**
40.0	0.0 (0)	15.0 (41)	24.0 (46)	12.0 (23)	21.0 (58)	29.0 (81)	26.0 (60)	16.0 (37)	34.0 (87)	36.0 (92)	14.0 (33)
80.0	0.0 (0)	11.0 (30)	21.0 (40)	11.0 (21)	20.0 (56)	24.0 (67)	**22.0 (51)**	18.0 (42)	27.0 (69)	29.0 (74)	14.0 (33)

^a^Medium + Cells; ^b^Medium + Cells + 0.2% DMSO (dimethyl sulphoxide); ^c^Percentage of control values are in parentheses

Boldfaced values signify the most effective doses

**Table 5:** Dose-response of anticancer properties of various compounds in breast cancer cells

Concentrations	Thiostrepton	Ampicillin	Dexamethasone	2-Methoxyestradiol	δ-Tocotrienol	(−) Riboflavin	Ascorbic Acid	Quercetin	Amiloride-HCL	(−) Corey Lactone	Quinine Sulphate
in μΜ	1	2	3	4	5	6	7	8	9	10	11
MC^a^ 0.0	17.0	17	17.0	17.0	18	20	20	20	20	20	44.0
MC + DMSO^b^ 0.0	23.0 (100)^c^	23.0 (100)	24.0 (100)	24.0 (100)	19.0 (100)	22.0 (100)	22.0 (100)	22.0 (100)	24.0 (100)	23.0 (100)	45.0 (100)
2.5	18.0 (78)	21.0 (91)	22.0 (92)	21.0 (88)	17.0 (89)	21.0 (95)	17.5 (80)	20.0 (91)	25.0 (104)	24.0 (104)	41.0 (91)
5.0	**10.0 (43)**	20.0 (90)	23.0 (96)	38.0 (79)	16.0 (84)	18.0 (82)	15.3 (70)	16.0 (73)	25.0 (104)	21.0 (91)	38.0 (84)
10.0	8.0 (35)	17.0 (74)	21.0 (88)	32.0 (67)	13.0 (68)	16.0 (73)	12.8 (58)	**8.0 (36)**	23.0 (96)	19.0 (91)	36.0 (80)
20.0	2.0 (9)	16.0 (70)	19.0 (79)	30.0 (63)	**8.0 (42)**	18.0 (82)	11.4 (52)	6.0 (27)	19.0 (79)	22.0 (96)	38.0 (84)
40.0	1.0 (4)	15.0 (65)	20.0 (83)	**20.0 (42)**	6.0 (32)	13.0 (59)	8.7 (40)	6.0 (27)	13.0 (54)	22.0 (96)	40.0 (89)
80.0	1.0 (4)	15.0 (65)	20.0 (83)	15.0 (31)	2.0 (11)	15.0 (68)	8.2 (37)	7.0 (32)	16.0 (67)	20.0 (87)	40.0 (89)

^a^Medium + Cells; ^b^Medium + Cells + 0.2% DMSO (dimethyl sulphoxide); ^c^Percentage of control values are in parentheses

Boldfaced values signify the most effective doses

Ampicillin, dexamethasone, (−) riboflavin, ascorbic acid, (−) Corey lactone and quinine sulphate were not effective anticancer agents in breast cancer (Table 5). Thiostrepton, dexamethasone, 2-methoxyestradiol, δ-tocotrienol, (−) riboflavin, ascorbic acid, and (−) Corey lactone were effective anticancer agents in the doses of 2.5 μM – 10 μM induced > 50% cancer cells apoptosis compared to respective controls in lung (Table 6). Most of the compounds in present group showed anticancer properties in cancer cells of lung (Table 6).

**Table 6:** Dose-response of anticancer properties of various compounds in lung cancer cells

Concentrations	Thiostrepton	Ampicillin	Dexamethasone	2-Methoxyestradiol	δ-Tocotrienol	(−) Riboflavin	Ascorbic Acid	Quercetin	Amiloride-HCL	(−) Corey Lactone	Quinine Sulphate
in μΜ	1	2	3	4	5	6	7	8	9	10	11
MC^a^ 0.0	12.0	24	17.0	17.0	17	32	32	32	20	21	46.0
MC + DMSO^b^ 0.0	17.0 (100)^c^	26.0 (100)^c^	22.0 (100)^c^	22.0 (100)^c^	26.0 (100)	33.0 (100)	33.0 (100)	33.0 (100)	27.0 (100)	27.0 (100)	43.0 (100)^c^
2.5	10.0 (59)	19.0 (73)	15.0 (68)	12.0 (54)	17.0 (65)	**9.0 (27)**	30.0 (91)	28.0 (85)	25.0 (93)	20.0 (74)	41.0 (95)
5.0	**7.0 (41)**	14.0 (54)	**10.0 (45)**	**8.0 (36)**	15.0 (58)	8.0 (24)	25.0 (76)	20.0 (61)	22.0 (82)	21.0 (78)	38.0 (88)
10.0	3.0 (18)	**12.0 (46)**	8.0 (36)	6.0 (27)	**11.0 (42)**	8.0 (24)	23.0 (70)	18.0 (55)	17.0 (63)	18.0 (67)	36.0 (84)
20.0	1.0 (6)	10.0 (38)	8.0 (36)	4.0 (18)	10.0 (38)	3.0 (9)	18.0 (55)	**16.0 (48)**	15.0 (56)	20.0 (74)	38.0 (88)
40.0	0.0 (0)	8.0 (31)	5.0 (23)	2.0 (9)	7.0 (27)	4.0 (12)	**16.0 (48)**	17.0 (52)	17.0 (63)	18.0 (67)	40.0 (93)
80.0	0.0 (0)	9.0 (35)	4.0 (18)	0.0 (0)	7.0 (27)	3.0 (9)	11.0 (33)	14.0 (42)	16.0 (59)	15.0 (56)	40.0 (93)

^a^Medium + Cells; ^b^Medium + Cells + 0.2% DMSO (dimethyl sulphoxide); ^c^Percentage of control values are in parentheses

Boldfaced values signify the most effective doses

### Dose-dependent response of anticancer properties of various compounds in melanoma, B-lymphocyte, and T-cells (Jurkat) cancer cell lines

Thiostrepton, 2-methoxyestradiol, δ-tocotrienol, quercetin, (−) Corey lactone, and quinine sulphate indicated anticancer properties at 5 μM - 80 μM for 50% apoptosis of melanoma cells (Table 7). On the other hand, thiostrepton (2.5 μM), 2-methoxyestradiol (2.5 μM), δ-tocotrienol (20 μM), quercetin (10 μM), amiloride-HCL and (−) Corey lactone (40 μM) indicated that more than 50% apoptosis compared to their respective controls with B lymphocytes (Table 8).

**Table 7:** Dose-response of anticancer properties of various compounds in melanoma cells

Concentrations	Thiostrepton	Ampicillin	Dexamethasone	2-Methoxyestradiol	δ-Tocotrienol	(−) Riboflavin	Ascorbic Acid	Quercetin	Amiloride-HCL	(−) Corey Lactone	Quinine Sulphate
in μΜ	1	2	3	4	5	6	7	8	9	10	11
MC^a^ 0.0	12.0	12.0	12.0	12.0	24.0	23.0	23.0	40.0	27.0	26.0	27.0
MC + DMSO^b^ 0.0	17.0 (100)^c^	17.0 (100)^c^	17.0 (100)^c^	17.0 (100)^c^	19.0 (100)	29.0 (100)	29.0 (100)	36.0 (100)	30.0 (100)	30.0 (100)	30.0 (100)^c^
2.5	10.0 (59)	15.0 (88)	41.0 (95)	9.0 (53)	17.0895)	21.0 (72)	26.0 (90)	32.0 (89)	28.0 (93)	27.0 (90)	29.0 (97)
5.0	**8.0 (47)**	13.0 (76)	38.0 (88)	**6.0 (35)**	14.0 (74)	16.0 (55)	22.0 (76)	27.0 (75)	25.0 (83)	24.0 (80)	26.0 (87)
10.0	6.0 (35)	12.0 (71)	36.0 (84)	2.0 (12)	**8.0 (42)**	**14.0 (48)**	23.0 (79)	22.0 (61)	22.0 (73)	27.0 (87)	28.0 (93)
20.0	3.0 (18)	10.0 (59)	38.0 (88)	1.0 (6)	5.0 (26)	12.0 (41)	22.0 (76)	**16.0 (44)**	25.0 (83)	20.0 (67)	28.0 (93)
40.0	4.0 (24)	9.0 (53)	40.0 (93)	0.0 (0)	2.0 (11)	12.0 (41)	19.0 (66)	8.0 (22)	27.0 (90)	17.0 (57)	25.0 (83)
80.0	2.0 (12)	9.0 (53)	40.0 (93)	0.0 (0)	07.0 (0)	13.0 (45)	20.0 (69)	5.0 (14)	26.0 (87)	**12.0 (47)**	26.0 (87)

^a^Medium + Cells; ^b^Medium + Cells + 0.2% DMSO (dimethyl sulphoxide); ^c^Percentage of control values are in parentheses

Boldfaced values signify the most effective doses

**Table 8:** Dose-response of anticancer properties of various compounds in B-lymphocytes cells

Concentrations	Thiostrepton	Ampicillin	Dexamethasone	2-Methoxyestradiol	δ-Tocotrienol	(−) Riboflavin	Ascorbic Acid	Quercetin	Amiloride-HCL	(−) Corey Lactone	Quinine Sulphate
in μΜ	1	2	3	4	5	6	7	8	9	10	11
MC^a^ 0.0	53.0	53	18.0	18.0	20	20	20	20	23	23	23.0
MC + DMSO^b^ 0.0	57.0 (100)^c^	57.0 (100)	23.0 (100)^c^	23.0 (100)^c^	23.0 (100)	23.0 (100)	23.0 (100)	23.0 (100)	25.0 (100)	25.0 (100)	26.0 (100)^c^
2.5	31.0 (54)	49.0 (86)	18.0 (78)	12.0 (52)	16.0 (70)	20.0 (87)	21.0 (91)	20.0 (87)	18.0 (72)	22.0 (74)	24.0 (92)
5.0	**18.0 (32)**	46.0 (81)	13.0 (56)	**8.0 (35)**	12.0 (52)	18.0 (78)	19.0 (83)	17.0 (74)	17.0 (68)	18.0 (61)	22.0 (85)
10.0	6.0 (11)	44.0 (77)	**9.0 (39)**	6.0 (26)	**10.0 (43)**	15.0 (65)	16.0 (70)	18.0 (78)	16.0 (64)	**14.0 (47)**	23.0 (88)
20.0	4.0 (7)	44.0 (77)	8.0 (35)	3.0 (13)	10.0 (43)	14.0 (61)	14.0 (61)	12.0 (52)	15.0 (60)	12.0 (41)	23.0 (88)
40.0	1.0 (2)	42.0 (74)	3.0 (13)	4.0 (17)	8.0 (35)	19.0 (83)	14.0 (61)	**10.0 (43)**	17.0 (68)	12.0 (41)	22.0 (85)
80.0	0.0 (0)	40.0 (70)	4.0 (17)	4.0 (17)	6.0 (26)	18.0 (78)	12.0 (52)	10.0 (43)	17.0 (68)	10.0 (34)	22.0 (85)

^a^Medium + Cells; ^b^Medium + Cells + 0.2% DMSO (dimethyl sulphoxide); ^c^Percentage of control values are in parentheses

Boldfaced values signify the most effective doses

**Fig. 2:**
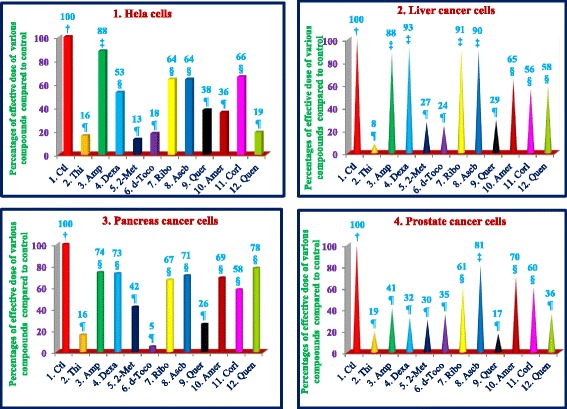
(1–4) Dose-dependent response for anti-proliferative properties of various compounds in cancer cells of Hela-1, liver-2, pancreas-3, and prostate-4. The cancer cell lines of Hela, liver, pancreas, and prostate were maintained in DMEM supplemented with 10% heat inactivated FBS and 10 mg/mL, gentamicin at 37 °C in a humidified atmosphere with 5% carbon dioxide (CO_2_) and 95% oxygen (O_2_) as described previously [17]. Cancer cells (1 × 10^5^) of various organs were seeded in 48 well tissue culture plate with 900 μl of medium containing 0.2% dimethyl sulfoxide of different types of cancer cell lines (Hela cell, liver, pancreas, and prostate), and incubated at 37 °C for 2 h. After 2 h, different concentrations (100 μl of 2.5, 5, 10, 20, 40, or 80 μM) of thiostrepton, ampicillin, dexamethasone, 2-methoxyestradiol, δ-tocotrienol, (−) riboflavin, ascorbic acid, quercetin, amiloride, and quinine sulphate in triplicate were added to each well, incubated for 48 h at 37 °C in a humidified atmosphere of 5% CO_2_. Followed by counting of live cells of each well by trypan blue dye exclusion or a quantitative colorimetric assay with 3-(4, 5)-dimethylthiozol-2, 5-diphenyl-tetrazolium bromide (MTT), as described previously [18, 19]. The anticancer properties and dose-dependence for eleven compounds are presented for Hela, liver, pancreas, and prostate cancer cell lines. Values in a column not sharing a common symbol are significantly different at ¶ = *P* < 0.001; § = *P* < 0.01; ‡ = *P* < 0.05; † = control

**Fig. 3:**
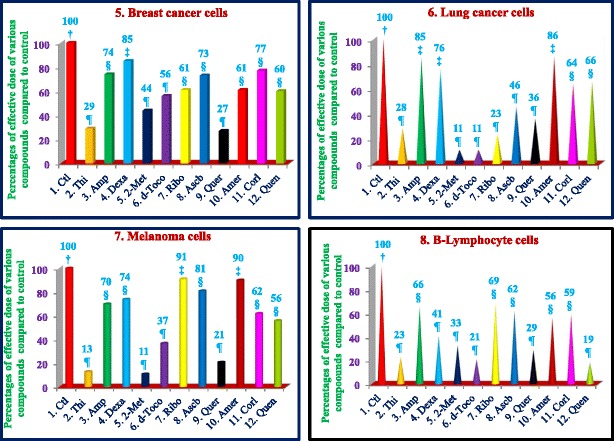
(5–8) Dose-dependent response for anti-proliferative properties of various compounds in cancer cell lines of breast-5, lung-6, Melanoma-7, and B-lymphocytes-8. The cancer cell lines of breast, lung, melanoma, and B-lymphocytes were maintained in DMEM supplemented with 10% heat inactivated FBS and 10 mg/mL, gentamicin at 37 °C in a humidified atmosphere with 5% carbon dioxide (CO_2_) and 95% oxygen (O_2_) as described previously [17]. Cancer cells (1 × 10^5^) of various organs were seeded in 48 well tissue culture plate with 900 μl of medium containing 0.2% dimethyl sulfoxide of different type of cancer cell lines (breast, lung, melanoma, and B-lymphocytes), and incubated at 37 °C for 2 h. After 2 h, different concentrations (100 μl of 2.5, 5, 10, 20, 40, or 80 μM) of thiostrepton, ampicillin, dexamethasone, 2-methoxyestradiol, δ-tocotrienol, (−) riboflavin, ascorbic acid, quercetin, amiloride, and quinine sulphate in triplicate added in each well, and then incubated for 48 h at 37 °C in a humidified atmosphere of 5% CO_2_. Followed by counting the living cells of each well by trypan blue dye exclusion or a quantitative colorimetric assay with 3-(4, 5)-dimethylthiozol-2, 5-diphenyl-tetrazolium bromide (MTT) as described previously [18, 19]. The anticancer properties as dose-dependent of eleven compounds presented for breast, lung, melanoma, and B-lymphocytes cancer cell lines. Values in a column not sharing a common symbol are significantly different at ¶ = *P* < 0.001; § = *P <* 0.01; ‡ = *P* ***<*** 0.05; † = control

**Fig. 4:**
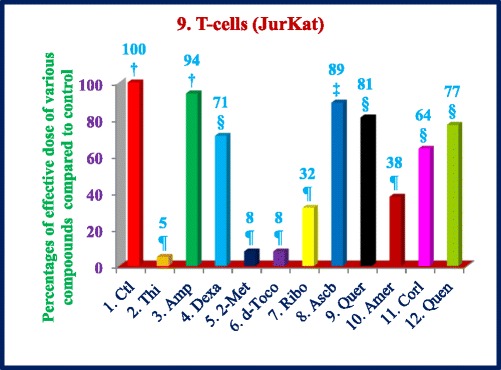
(9) Dose-dependent response for anti-proliferative properties of various compounds in T-cells (Jurkat). The T-cells (Jurkat) maintained in DMEM supplemented with 10% heat inactivated FBS and 10 mg/mL, gentamicin at 37 °C in a humidified atmosphere with 5% carbon dioxide (CO_2_) and 95% oxygen (O_2_) as described previously [17]. The T-cells (1 × 10^5^) of was seeded in 48 well tissue culture plate with 900 μl of medium, containing 0.2% dimethyl sulfoxide of T-cells (Jurkat), and incubated at 37 °C for 2 h. After 2 h, different concentrations (100 μl of 2.5, 5, 10, 20, 40, or 80 μM) of thiostrepton, ampicillin, dexamethasone, 2-methoxyestradiol, δ-tocotrienol, (−) riboflavin, ascorbic acid, quercetin, amiloride, and quinine sulphate in triplicate added in each well, and then incubated for 48 h at 37 °C, in a humidified atmosphere of 5% CO_2_. Followed by counting the living cells of each well by trypan blue dye exclusion or a quantitative colorimetric assay with 3-(4, 5)-dimethylthiozol-2, 5-diphenyl-tetrazolium bromide (MTT) as described previously [18, 19]. The anti-proliferation properties as dose-dependent with eleven compounds were presented in T-cells (Jurkat). Values in a column not sharing a common symbol are significantly different at ¶ = *P* < 0.001; § = *P* ***<*** 0.01; ‡ = *P* ***<*** 0.05; † = control

Similarly, thiostrepton, 2-methoxyestradiol (2.5 μM), δ-tocotrienol (20 μM), quercetin (10 μM), amiloride-HCL, (−) Corey lactone, and quinine sulphate (80 μM) induced at least 50% cancer cells apoptosis in T-cells (Jurkat) compared to their respective controls (Table 9). In summary, thiostrepton, 2-methoxyestradiol, δ-tocotrienol, and quercetin were clearly very effective and induced apoptosis in the range (70% - 92%) in Hela and liver cancer cells. Thus thiostrepton, dexamethasone, 2-methoxyestradiol, δ-tocotrienol, quercetin, and quinine sulfate have potent anticancer properties in pancreas, prostate, breast, lungs, melanoma, B-lymphocytes, and T-cells (Jurkat) in the range of 40% to 80% compared to respective controls. The most effective dose for anticancer properties for all eleven compounds in each organ were compared to respective controls (Table 10; Fig. 2 (1–4) and Fig. 3 (5–8)). The significant decreases (64% - 84%) were noted in anticancer activity, and most effective doses were (20 μM – 80 μM) of thiostrepton, 2-methoxyestradiol, δ-tocotrienol, quercetin, amiloride-HCL in Hela cells as shown in Fig. 2. Thiostrepton, 2-methoxyestradiol, δ-tocotrienol, and quercetin caused > 50% anticancer activity in liver and pancreatic cancer cells (Fig. 2). Whereas, thiostrepton, ampicillin, dexadexamethasone, 2-methoxyestradiol, δ-tocotrienol, quercetin, and quinine sulphate treatment induced cell death more than 50% in prostate cancer cells (Fig. 2). In breast cancer cells, only thiostrepton, 2-methoxyestradiol, and quercetin induced more than 50% cell apoptosis (Fig. 3). The anticancer activities resulted in > 50% with thiostrepton, 2-methoxyestradiol, δ-tocotrienol, riboflavin, ascorbic acid and quercetin in lung cancer cells (Fig. 3). There were more than 50% anticancer activities noted with treatment of thiostrepton, 2-methoxyestradiol, δ-tocotrienol, quercetin in melanoma cells (Fig. 3); thiostrepton, dexamethasone, 2-methoxyestradiol, δ-tocotrienol, quercetin, quennine sulphate in B-lymphocytes cells (Fig. 3); and thiostrepton, 2-methoxyestradiol, δ-tocotrienol, riboflavin in T-cells (Jurkat, Fig. 4). These results were translated possible IC_50_ values of anticancer activities as shown in Table 11. These findings further supported by calculation of log IC_50_ values from original data of Tables 1–9 by GraphPad Prism 5 program.

**Table 9:** Dose-response of anticancer properties of various compounds in T-cells (Jurkat)

Concentrations	Thiostrepton	Ampicillin	Dexamethasone	2-Methoxyestradiol	δ-Tocotrienol	(−) Riboflavin	Ascorbic Acid	Quercetin	Amiloride-HCL	(−) Corey Lactone	Quinine Sulphate
in μΜ	1	2	3	4	5	6	7	8	9	10	11
MC^a^ 0.0	42.0	42	36.0	36.0	40	40	32	36	41	42	42.0
MC + DMSO^b^ 0.0	47.0 (100)^c^	47.0 (100)^c^	39.0 (100)^c^	39.0 (100)	44.0 (100)	44.0 (100)	33.0 (100)	40.0 (100)	44.0 (100)	46.0 (100)	48.0 (100)^c^
2.5	**21.0 (45)**	45.0 (96)	34.0 (87)	21.0 (54)	40.0 (91)	42.0 (95)	29.0 (88)	34.0 (85)	41.0 (93)	42.0 (91)	46.0 (96)
5.0	18.0 (38)	44.0 (93)	30.0 (77)	**18.0 (46)**	35.0 (80)	40.0 (91)	27.0 (82)	29.0 (73)	39.0 (89)	38.0 (83)	44.0 (92)
10.0	10.0 (21)	42.0 (91)	26.0 (67)	16.0 (41)	**22.0 (50)**	39.0 (89)	25.0 (74)	**18.0 (45)**	37.0 (84)	34.0 (74)	41.0 (85)
20.0	8.0 (17)	41.0 (87)	28.0 (72)	8.0 (21)	19.0 (43)	36.0 (82)	24.0 (73)	13.0 (33)	33.0 (75)	31.0 (67)	38.0 (79)
40.0	3.0 (6)	40.0 (85)	**25.0 (24)**	3.0 (8)	13.0 (30)	39.0 (89)	24.0 (73)	10.0 (25)	32.0 (73)	29.0 (63)	40.0 (83)
80.0	3.0 (6)	41.0 (87)	24.0 (62)	3.0 (8)	10.0 (23)	36.0 (82)	21.0 (64)	7.0 (18)	32.0 (73)	27.0 (59)	40.0 (83)

^a^Medium + Cells; ^b^Medium + Cells + 0.2% DMSO (dimethyl sulphoxide); ^c^Percentage of control values are in parentheses

Boldfaced values signify the most effective doses

**Table 10:** Impact of effective dose of various compounds in different cancer cell lines

#		Hela cells	Liver cancer cells	Pancreas cancer cells	Prostate cancer cells	Breast cancer cells	Lung cancer cells	Melanoma cells	B-Lymphocyte cells	T-cells (Jurkat)
		1	2	3	4	5	6	7	8	9
	Compounds	μM; value (%)^a^	μM; value (%)	μM; value (%)	μM; value (%)	μM; value (%)	μM; value (%)	μM; value (%)	μM; value (%)	μM; value (%)
1	Thiostrepton	40; 8.7 ± 1.5 (16)^a^	10; 6.7 ± 2.2 (8)	10; 7.7 ± 0.6 (16)	5; 7.0 ± 1.7 (19)	10; 6.7 ± 0.6 (29)	5; 7.0 ± 2.0 (28)	5; 2.3 ± 1.5 (13)	2.5; 15.7 ± 4.0 (23)	2.5; 21.3 ± 2.1 (5)
2	Ampicillin	80; 47.7 ± 5.5 (88)	40; 70.3 ± 4.2 (88)	80; 35.3 ± 5.0 (74)	20; 15.0 ± 1.0 (41)	40; 16.7 ± 6.1 (74)	80; 21.3 ± 1.5 (85)	20; 12.3 ± 1.2 (70)	10; 44.7 ± 5.1 (66)	20; 450.3 ± 15.6 (94)
3	Dexamethasone	80; 18.0 ± 4.0 (53)	80; 67.3 ± 4.0 (93)	20; 34.7 ± 4.0 (73)	20; 16.7 ± 1.5 (32)	80; 15.7 ± 3.1 (85)	20; 16.7 ± 2.1 (76)	40; 13.0 ± 2.7 (74)	10; 61.0 ± 3.6 (41)	40; 282.0 ± 21.7 (71)
4	2-Methoxyestradiol	20; 4.3 ± 1.5 (13)	10; 19.7 ± 2.1 (27)	40; 20.0 ± 2.0 (42)	10; 1.7 ± 1.5 (30)	20; 8.0 ± 2.0 (44)	10; 2.3 ± 1.5 (11)	10; 2.0 ± 1.7 (11)	5; 4.0 ± 2.0 (33)	5; 31.3 ± 5.7 (8)
5	δ-Tocotrienol	20; 8.3 ± 2.1 (18)	20; 17.7 ± 2.1 (24)	20; 5.7 ± 1.5 (5)	20; 12.3 ± 1.5 (35)	20; 10.7 ± 0.6 (56)	20; 8.3 ± 2.1 (11)	20; 7.0 ± 3.6 (37)	20; 2.7 ± 1.5 (21)	5; 31.3 ± 5.7 (8)
6	(−) Riboflavin	40; 21.0 ± 1.0 (64)	80; 74.0 ± 2.0 (91)	40; 40.0 ± 2.0 (67)	40; 20.0 ± 2.0 (61)	80; 12.7 ± 1.5 (61)	10; 7.7 ± 2.1 (23)	40; 26.3 ± 3.1 (91)	20; 19.3 ± 3.1 (69)	20; 142.0 ± 4.4 (32)
7	Ascorbic Acid	80; 21.0 ± 0.1 (64)	80; 74.3 ± 2.5 (90)	80; 42.3 ± 2.5 (71)	80; 29.3 ± 2.1 (81)	40; 15.0 ± 1.7 (73)	20; 15.0 ± 4.4 (46)	80; 23.7 ± 4.7 (81)	20; 17.3 ± 2.5 (62)	40; 451.3 ± 32.4 (89)
8	Quercetin	40; 21.3 ± 1.5 (38)	40; 24.0 ± 2.0 (29)	40; 11.7 ± 0.6 (26)	20; 7.3 ± 1.2 (17)	20; 5.7 ± 0.6 (27)	20; 11.7 ± 1.5 (36)	40; 8.3 ± 0.6 (21)	10; 8.0 ± 3.0 (29)	40; 409.3 ± 6.4 (81)
9	Amiloride-HCL	80; 19.7 ± 1.5 (36)	40; 36.7 ± 2.5 (65)	80; 30.7 ± 1.2 (69)	80; 27.0 ± 4.6 (70)	20; 14.0 ± 5.3 (61)	40; 23.3 ± 2.5 (86)	40; 33.0 ± 5.6 (90)	20; 5.0 ± 1.0 (56)	20; 132.7 ± 2.1 (38)
10	(−) Corey Lactone	80; 36.0 ± 5.6 (66)	80; 31.7 ± 2.1 (56)	40; 30.7 ± 1.2 (58)	40; 23.3 ± 2.1 (60)	40; 17.7 ± 3.2 (77)	40; 17.3 ± 3.8 (64)	40; 18.3 ± 3.5 (62)	20; 5.3 ± 1.5 (59)	40; 280.3 ± 10.2 (64)
11	Quinine Sulphate	20; 10.7 ± 1.5 (19)	80; 47.7 ± 2.1 (58)	40; 35.0 ± 3.0 (78)	20; 15.7 ± 2.1 (36)	80; 12.3 ± 2.3 (60)	40; 21.3 ± 1.5 (66)	20; 22.7 ± 2.9 (56)	80; 20.3 ± 3.1 (19)	40; 267.7 ± 29.7 (77)

^a^Percentage of control values are in parentheses

**Table 11:** The IC50 values of various compounds in different cancer cell lines

#		Hela cells	Liver cancer cells	Pancreas cancer cells	Prostate cancer cells	Breast cancer cells	Lung cancer cells	Melanoma cells	B-Lymphocyte cells	T-cells (Jurkat)
		1	2	3	4	5	6	7	8	9
	Compounds	μM; value (%)^a^	μM; value (%)	μM; value (%)	μM; value (%)	μM; value (%)	μM; value (%)	μM; value (%)	μM; value (%)	μM; value (%)
1	Thiostrepton	10; 13.3 ± 0.6 (24)^a^	2.5; 42.3 ± 3.5 (53)	5; 14.0 ± 1.0 (29)	2.5; 19.7 ± 0.6 (53)	20; 3.7 ± 1.5 (16)	5; 7.0 ± 2.0 (48)	2.5; 6.0 ± 2.0 (34)	2.5; 15.7 ± 4.0 (23)	2.5; 21.3 ± 2.1 (5)
2	Ampicillin				5; 18.3 ± 1.5 (50)					
3	Dexamethasone	20; 18.3 ± 4.2 (54)			2.5; 23.3 ± 2.0 (45)		2.5; 10.3 ± 2.1 (47)		5; 66.0 ± 5.3 (45)	
4	2-Methoxyestradiol	2.5; 16.3 ± 1.5 (48)	5; 32.7 ± 2.5 (45)	40; 20.0 ± 2.0 (42)	2.5; 20.0 ± 2.0 (39)	10; 8.3 ± 2.9 (45)	2.5; 3.7 ± 1.2 (17)	2.5; 4.3 ± 0.6 (25)	2.5; 8.0 ± 2.0 (5)	2.5; 32.7 ± 5.0 (8)
5	δ-Tocotrienol	5; 20.7 ± 1.2 (45)	20; 38.3 ± 5.9 (53)	10; 18.0 ± 2.0 (36)	10; 14.0 ± 2.0 (39)	10; 9.3 ± 2.1 (49)	20; 2.3 ± 2.1 (11)	10; 9.3 ± 3.1 (50)	10; 7.0 ± 2.1 (55)	20; 142.0 ± 4.4 (32)
6	(−) Riboflavin				80; 18.0 ± 2.0 (55)		2.5; 9.3 ± 2.5 (28)		80; 15.3 ± 3.5 (55)	
7	Ascorbic Acid						10; 16.0 ± 4.6 (49)			
8	Quercetin	10; 28.3 ± 8.1 (51)	20; 26.3 ± 3.5 (32)	40; 20.3 ± 2.1 (45)	10; 21.0 ± 3.6 (48)	10; 8.0 ± 1.7 (39)	10; 14.7 ± 2.5 (45)	10; 21.7 ± 7.1 (54)	10; 8.0 ± 3.0 (29)	10; 157.7 ± 20.5 (45)
9	Amiloride-HCL	10; 27.7 ± 2.1 (51)	80; 31.7 ± 2.1 (56)			40; 12.0 ± 2.0 (52)			40; 4.3 ± 1.5 (48)	
10	(−) Corey Lactone		80; 31.7 ± 2.1 (56)	80; 28.7 ± 3.1 (54)	80; 19.7 ± 1.5 (51)	80; 12.7 ± 1.5 (55)	80; 12.0 ± 2.7 (44)	80; 13.7 ± 2.3 (46)	40; 3.3 ± 1.5 (37)	80; 124.3 ± 17.4 (29)
11	Quinine Sulphate	10; 15.3 ± 2.5 (24)	80; 47.7 ± 2.1 (58)		20; 15.7 ± 2.1 (36)			80; 20.3 ± 5.0 (50)		80; 174.7 ± 8.1 (50)

^a^Percentage of control values are in parentheses

**Fig. 5:**
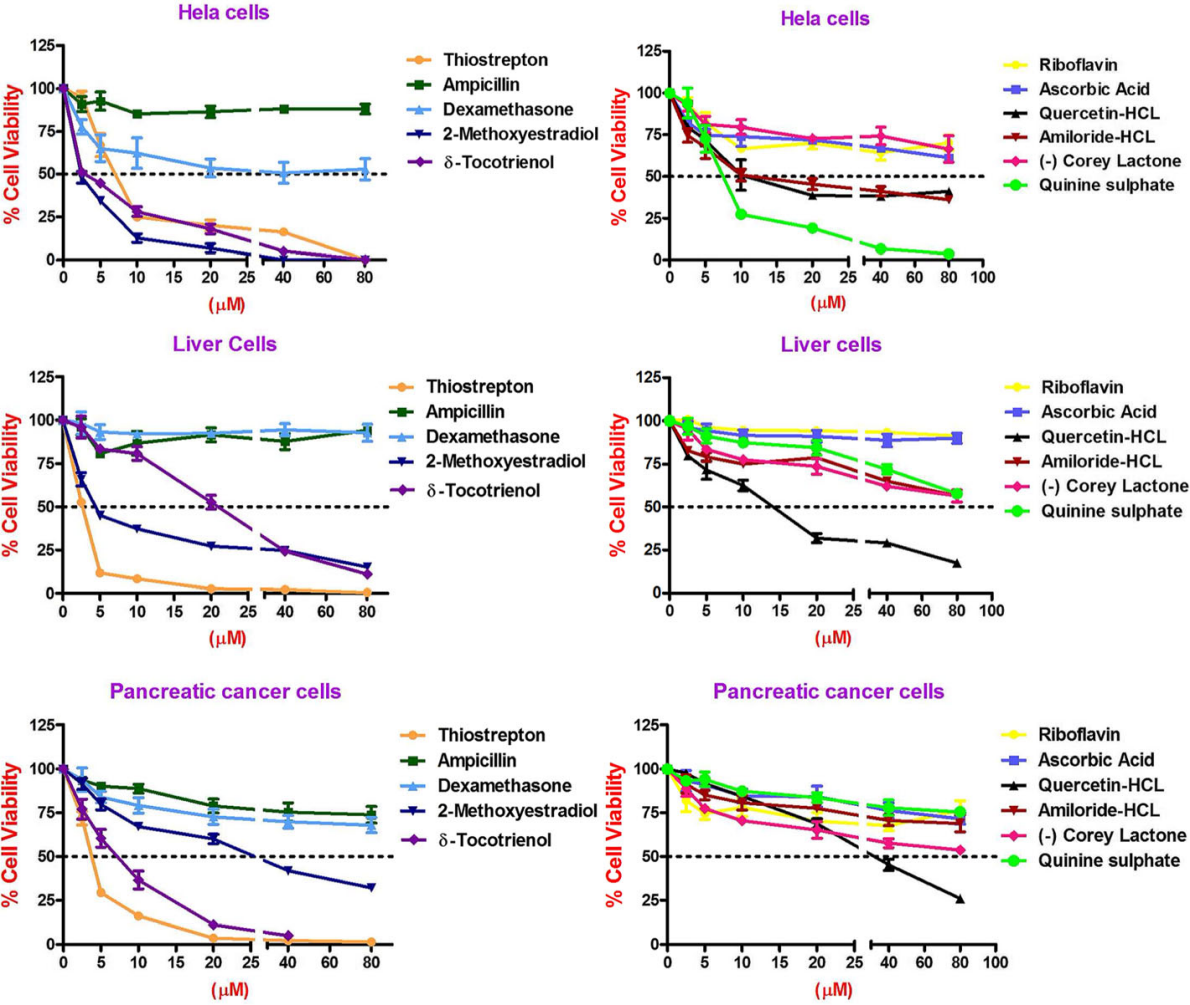
The log IC_50_ values for anti-proliferative properties of eleven compounds in Hela cells, liver, and pancreas cancer cells. The log IC_50_ values calculated from original data of Tables 1-9 by GraphPad Prism 5 program. The left hand side figure shows the log IC_50_ of first five compounds (thiostrepton, ampicillin, dexamethasone, 2-methoxyestradiol, δ-tocotrienol, and right hand side figure indicates the remaining six compounds [(−) riboflavin, ascorbic acid, quercetin, amiloride, and quinine sulphate] of Hela, liver and pancreas cancer cells

**Fig. 6:**
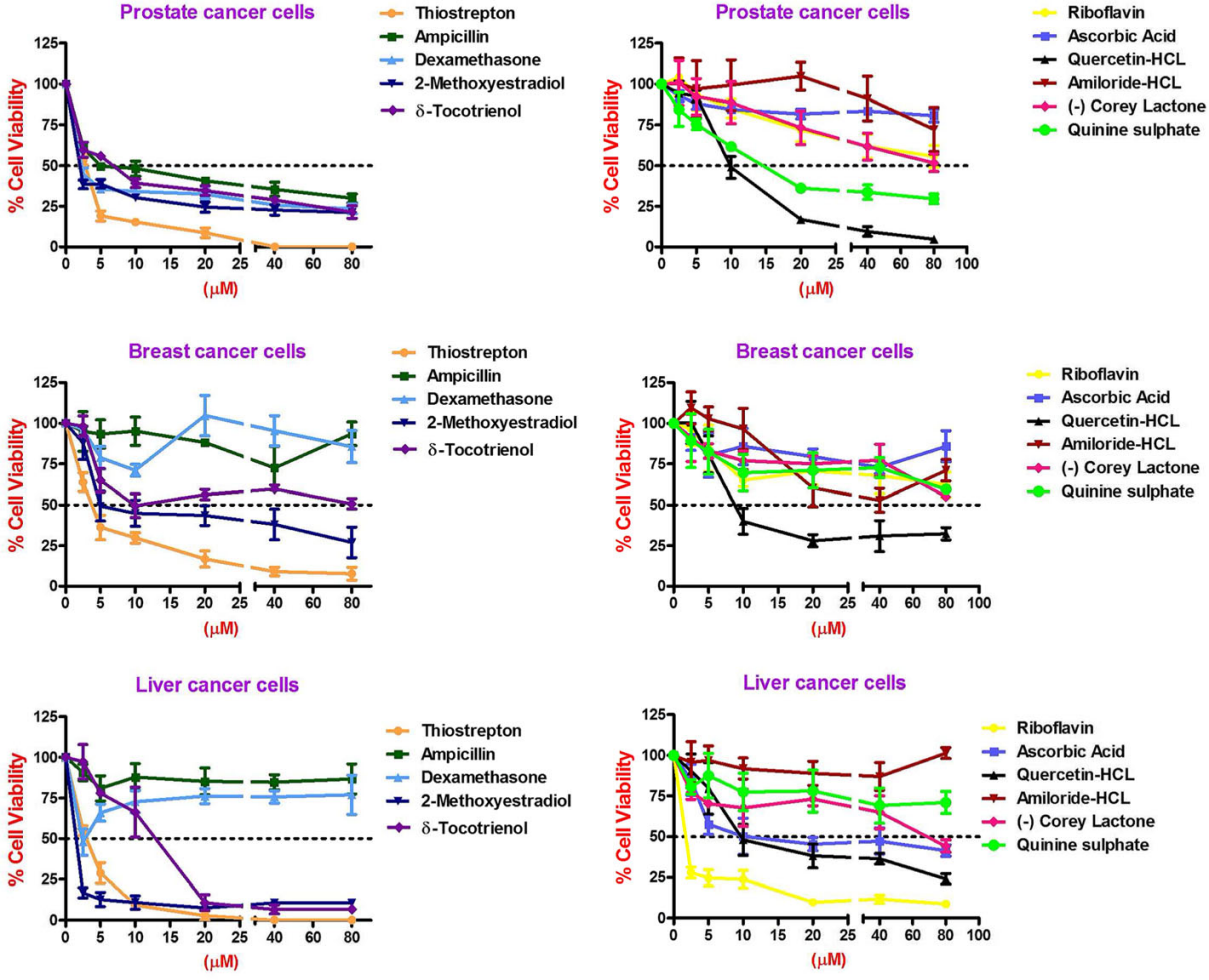
The log IC_50_ values for anti-proliferation properties of eleven compounds in prostate, breast and lung cancer cells. The log IC_50_ values calculated from original data of Tables 1-9 by GraphPad Prism 5 program. The left hand figure showed the logIC_50_ of first five compounds (thiostrepton, ampicillin, dexamethasone, 2-methoxyestradiol, δ-tocotrienol, and right hand figure indicated the remaining six compounds ((−) riboflavin, ascorbic acid, quercetin, amiloride, and quinine sulphate) of prostate, breast and lung cancer cells

**Fig. 7:**
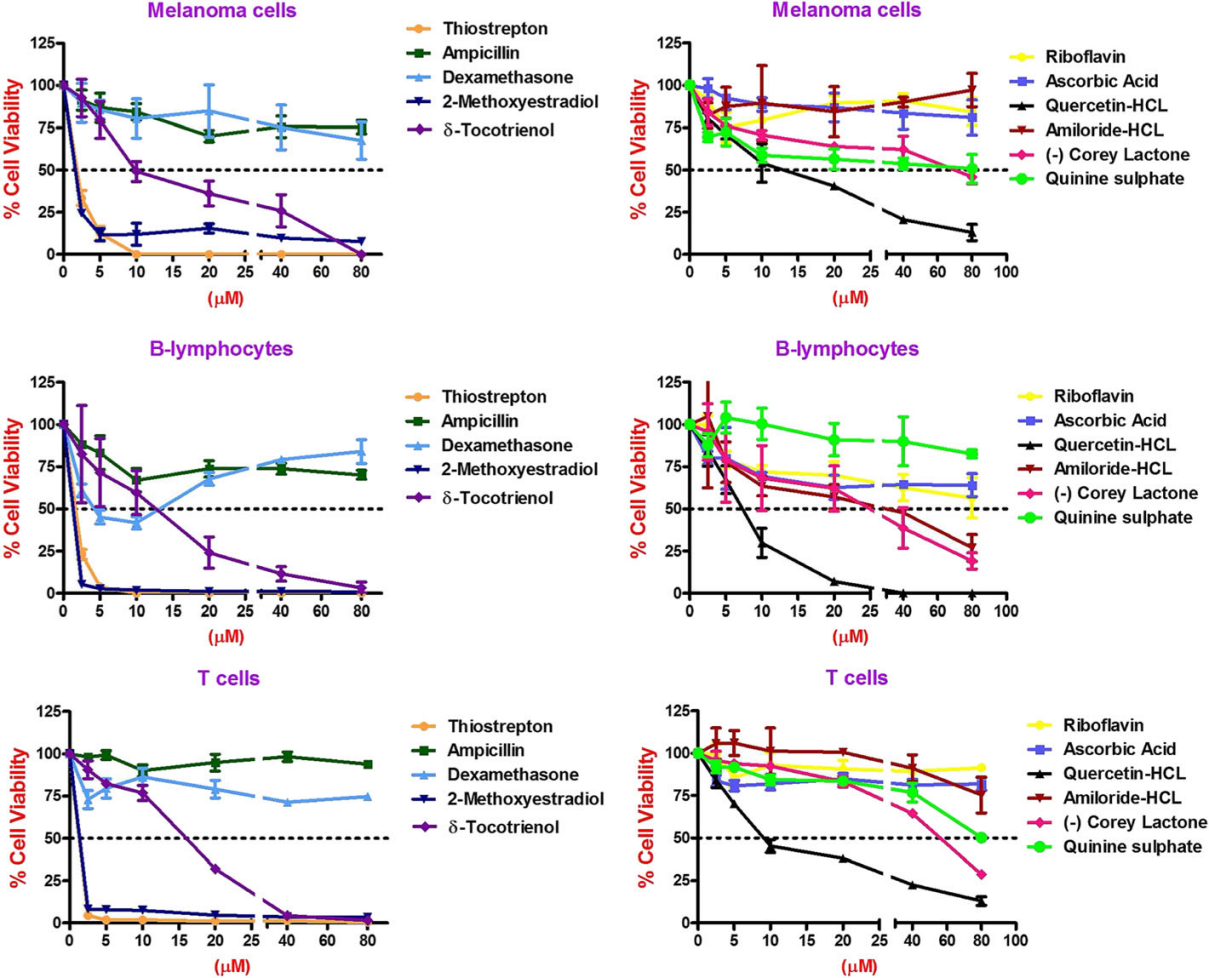
The log IC_50_ values of anti-proliferation properties of eleven compounds in melanoma, B-lymphocytes, and T-cells. The log IC_50_ values calculated from original data of Tables 1-9 by GraphPad Prism 5 program. The left hand side figure showed the log IC_50_ of first five compounds (thiostrepton, ampicillin, dexamethasone, 2-methoxyestradiol, δ-tocotrienol, and right hand side figure indicates the remaining six compounds ((−) riboflavin, ascorbic acid, quercetin, amiloride, and quinine sulphate) of melanoma, B-lymphocytes, and T-cells

The log IC_50_ for anticancer properties of each compound in each organ of cancer cells calculated for doses of 2.5 μM to 80 μM by GraphPad Prism 5 (Table 11). The use of present doses of 2.5 μM to 80 μM resulted log IC_50_ for only thiostrepton, 2-methoxyestradiol, δ-tocotrienol, and quercetin for each organ of cancer cells as shown in Table 12. The addition of higher doses of 160 μM and 320 μM would have provided logIC_50_ for all the compounds for cancer cells of each organ as evident from Tables 1–9, and graphs of GraphPad Prism 5 (Figs. 5, 6 and 7). Apart from thiostrepton, 2-methoxyestradiol, δ-tocotrienol, and quercetin, most of the rest of compounds indicated anticancer properties between 51% - 58% with doses of 40 μM and 80 μM in all organs tested.

**Table 12:** The logIC50 values of anticancer properties of various compounds in diffenent cancer cell lines

	Compounds	Thiostrepton	Ampicillin	Dexamethasone	2-Methoxyestradiol	δ -Tocotrienol	(−) Riboflavin	Ascorbic Acid	Quercetin	Amiloride-HCL	(−) Corey Lactone	Quinine Sulphate
	Cancer Cell lines	1	2	3	4	5	6	7	8	9	10	11
1	Hela cells	7.27		65.44	3.37	5.17	−16,620,000.00	100.60	37.31	36.13	−18,170,000.00	7.68
2	Liver cancer cells	2.69			8.62	24.61		−7,318,000.00	18.05	86.77	81.82	
3	Pancreas cancer cells	3.87	−16,950,000.00	−19,140,000.00	42.31	7.70		−18,390,000.00	43.63	−18,160,000.00	−30,360,000.00	−16,470,000.00
4	Prostate cancer cells	2.91	23.49	7.92	5.01	14.96			10.63		74.50	25.60
5	Breast cancer cells	5.09	306.80	806.70	26.86	67.48		295.10	11.29		88.04	96.53
6	Lung cancer cells	3.31		− 311.50	1.92	12.19	1.95	43.51	24.49			
7	Melanoma cells	2.10		115.40	1.99	15.81		−13,670,000.00	17.02			65.25
8	B-Lymphocyte cells	2.06	140.00		1.91	12.64	81.84	−17,420,000.00	7.45	41.27	33.54	143.80
9	T-cells (Jurkat)	1.92	376.60	−10,530,000.00	1.88	16.00	391.10	−5,645,000.00	15.38		40.28

### Estimations of plasma cytokines of total mRNAs obtained after δ-tocotrienol treatment to hepatitis C patients

**Fig. 8:**
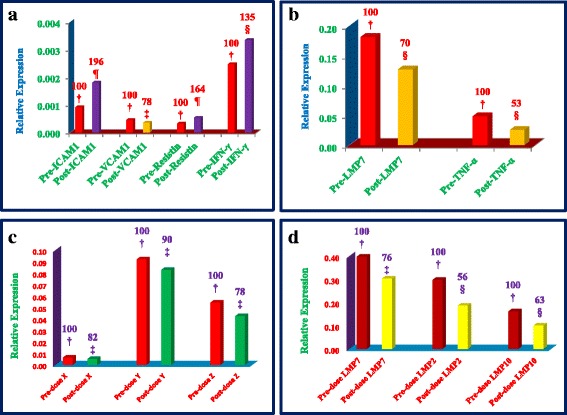
**a**-**d** The expression of important inflammatory biomarkers and proteasome subunits of total RNAs obtained from plasma after EDTA treated whole blood after feeding δ-tocotrienol (500 mg/d) for 6-weeks to hepatitis C patients. The quantitative Real-time qRT-PCR was performed on total mRNA isolated from plasma samples of hepatitis C patients of pre-dose and post-dose samples. Taqman primer-probes and Step-one qRT-PCR kit were used for these estimations. All reactions were performed in triplicate using equal amounts of total RNA per reaction. Reverse-transcriptase step involved incubation at 48 °C for 15 min. The PCR cycling conditions included an initial denaturation at 95 °C for 10 min, followed by 40 cycles of 95 °C for 15 s, and 60 °C for 60 s. Real-time PCR assays were completed using a Step-one plus Real time PCR system. Relative gene expression of hepatitis C patients of pre-dose vs post-dose samples were normalized (2^−ΔΔC^_T_ analysis) to GAPDH*.* Values in a column not sharing a common symbol are significantly different at ¶ = *P* < 0.001; § = *P <* 0.01; ‡ = *P <* 0.05; † = control

Since present in vitro data indicated that δ-tocotrienol is a very effective apoptosis-inducing agent amongst other outstanding potent compounds (thiostrepton, 2-methoxyestradiol, quercetin) for cancer cells apoptosis tested in all nine cell lines (Hela cells, liver, pancreas, prostrate, breast, breast, melanoma, B lymphocytes, and T-cells (Jurkat). We decided to test the efficiency of δ-tocotrienol in hepatitis C patients. δ-Tocotrienol (500 mg/d) was administered to hepatitis C patients (*n* = 5) for 6 weeks. The plasma values of alanine aminotransferase (ALT) of pre-dose versus post-dose of δ-tocotrienol treatment of hepatitis C patients showed significant decrease (12%) in post-dose values, compared to pre-dose values (Table 13). The plasma AST also decreased (14%) in post-dose values, compared to pre-dose values (Table 13). The total RNAs obtained from δ-tocotrienol treated hepatitis C patients showed significant decrease in expression of pro-inflammatory cytokines such as TNF-α (47%; ***P*** < 0.001), VCAM-1 (22%; ***P*** < 0.01). However, there was a robust increase in expression of pro-inflammatory cytokines such as ICAM-1 (96%), and IFN-γ (35%) after post-treatment (Fig. 8a, b). The expression of proteasome subunits X (18%), Y (10%) and Z (22%) was not affected significantly (because these proteasome subunits are not greatly expressed in hematopoietic cells) as compared to other proteasome subunits LMP7 (24%), LMP2 (44%), and LMP10 (37%; *P* < 0.001) decreased significantly as shown in Fig. 8c, d. These data suggest that expression of proteasome LMP subunits and TNF-α were significantly down regulated after δ-tocotrienol treatment. Down-regulation of proteasome subunits leads to autophagy and apoptosis of cells.

**Table 13:** Physical characteristics of hepatitis C patients

		Hepatitis C patients	
		Pre-dose values	Post-dose values
	Parameters	Median (IQR)^a^	Median (IQR)
1	Subjects		
	Male (*n*)	8	8
	Female (*n*)	6	6
	Total (males + female)	14	14
2	Age (years)	44 (39–50)	44 (39–50)
	Gender Male (%)	8 (57)	8 (57)
	Gender Female (%)	6 (43)	6 (43)
3	Weight (kg)^a^	69 (64–72)	68 (52–70)
4	Height (cm)	169 (162–171)	169 (162–171)
5	BMI (kg/m2)	24 (23–25)	23 (22–24)
6	Pulse/minte	71 (68–79)	73 (70–77)
7	Temperature (°C)	37.4 (37.2–38.3)	37.5 (37.3–37.8)
8	Systolic blood pressure (mmHg)	125 (122–131)	120 (118–129)
	Diastolic blood pressure (mmHg)	85 (80–90)	80 (75–85)
9	Heamoglobin (g/L)	142 (137–150)	143 (138–154)
10	Total Leukocytes counts (109/L)	5404 (4316–6505)	5385 (4523–7725)
11	Bilirubin (μmmol/L)	15 (11–22)	16 (10–20)
12	Alanine Aminotransferase (ALT; U/L)	68 (64–79)	60 (53–69)^b^
13	Aspartate Aminotransferase (AST; U/L)	56 (53–59)	48 (45–51)^b^
14	Alaline Phosphatase (ALP; U/L)	84 (72–91)	77 (68–85)^b^
15	Creatinine (μmol/L)	92 (84–109)	85 - (74–113)

^a^*IQR* Interquartile Range (25–75); ^b^Wilcoxon Sinf rank test applied *P* < 0.001

## Discussion

This is the first study, in which several natural compounds were compared for their anticancer properties in several different cancer organ cell lines (liver, pancreas, prostate, breast, lung) and cell lines (Hela, B-lymphocytes, melanoma, and T-cells). The present results clearly demonstrate the effectiveness of several naturally occurring and FDA approved compounds with anticancer properties in several organs of humans. Collectively, thiostrepton, dexamethasone, 2-methoxyestradiol, δ-tocotrienol and quercetin were very effective anticancer agents for Hela, liver, pancreas, prostate, breast, lung, melanoma, B-lymphocytes and T-cell lines (Jurkat). Although, the present investigation was carried out for anticancer activity of eleven compounds, the IC_50_ values were obtained for only four compounds, which was due to use of a small range of doses of 2.5 μM to 80 μM. These four compounds described above are effective anticancer properties for cancer cells for all organs of human. The other compounds (amiloride-HCL, (−) Corey lactone, quinine sulphate) were effective only in some cancer cell lines. Higher doses of 160 μM and 320 μM should have been included in the present study. However, these natural compounds can act at the proteasome level, inhibition of the proteasome’s activities leads to Autophagy, as the cancer cells cannot degrade ubiquitinated proteins.

Thiostrepton has two-fold mechanism of action, first as anti-intracellular infection (targeting bacterial parasites), and second, induces autophagy to enhance host cell defense system by activating endoplasmic reticulum (ER) stress pathways in eukaryotes [20]. This intracellular response is sensitive to the modification of the quinaldic acid, which is found in all the thiopeptide family of antibiotics. The quinaldic acid removes intracellular pathogens from the body. Therefore, thiostrepton has dual action on parasitic bacteria and infected host cells [20]. The role of autophagy is highly complex and context-dependent [21], leading to both cancer suppression and progression in several tumors as observed in the present study in breast, prostate, lung and melanoma cancers by inhibition of the proteasome’s proteases.

Blocking the proteasome’s proteases leads to autophagy. Autophagy is a conserved process that uses double-membrane vesicle to deliver cytoplasmic contents to lysosomes for degradation. Therefore, autophagy may affect many facets of human biology and disease. Autophagy also protects against certain neurodegenerative and infectious diseases [22]. Autophagy enhances the clearance of toxic, cytoplasmic, aggregate-prone proteins and infectious agents from the body. Therefore, beneficial roles of autophagy extended to supporting cell survival and regulating inflammation [22]. The degradation of protein is an essential cellular function that, when dys-regulated or impaired, can lead to wide variety of diseases [23]. In summary, there are two intracellular protein degradation systems known as ubiquitin-proteasome system and autophagy, which act as a catabolic process that involves delivery of cellular components to lysosomes for degradation. The recent studies have indicated interaction between ubiquitin-proteasome system and autophagy, and their coordinated and complementary relationship becomes critical in times of cellular stress [23]. These are two major intracellular protein degradation systems that work cooperatively to maintain homeostasis [24].

As mentioned earlier, that proteasome inhibitors have clinical activity in hematological tumors, and inhibitors of autophagy evaluated as potential antitumor therapies, and reported that proteasome inhibitors and small interfering RNA-mediated knockdown of the proteasome’s enzymatic subunits promoted auto phagosome formation as also observed in present study. The stimulated autophagy flux, and up-regulated expression of the autophagy-specific genes, ATG5 and ATG7 observed in some human prostate cancer cells [24]. The up-regulation of ATG5 and ATG7 occurred in cells displaying proteasome inhibitor-induced phosphorylation of the eukaryotic translation initiation factor 2 α (eIF2α), an important component of the unfolded protein responses. Moreover, proteasome inhibitors did not induce autophagy or up-regulate ATG5 in mouse embryonic fibroblasts expressing a phosphorylation-deficient mutant form of eIF2α [24]. Combined inhibition of autophagy and proteasome, induced an accumulation of intracellular protein aggregates reminiscent of neuronal inclusion bodies, and caused more cancer cell death than blocking either degradation pathway alone [24]. Overall, their data show that proteasome inhibition activates autophagy through a phosphor-eIF2α-dependent mechanism to eliminate protein aggregates and alleviate proteotoxic stress in human prostate cancer cells [24]. Moreover, autophagy in prostate cancer occurred during androgen deprivation, and an attractive possibility of autophagy inhibition combined with hormonal therapy is a promising approach for prostate cancer treatment [21].

Ampicillin is a broad spectrum antibiotic and used to treat respiratory tract infections, urinary infections, salmonellosis and meningitis. It is effective against both Gram-positive and Gram-negative bacteria. It acts as an irreversible inhibitor of the enzyme *trans*-peptidase, which needed by bacteria to make their cell wall [25]. It inhibits in final stage of cell wall synthesis in binary fission, leads to cell lysis, thus known as bacteriolytic [25, 26]. Dexamethasone used as anti-inflammatory agent to treat rheumatoid arthritis, bronchospasm, and bacterial meningitis. It is also used to counteract some side effects of some chemotherapy drugs in cancer patients, particularly with brain tumor to counteract the development of edema [27]. Riboflavin is involved in converting food (carbohydrate) into fuel (glucose), which is used to produce energy. It is also involved in metabolizing body fats and protein. It is required for proper healthy liver, skin, hair, eyes and the nervous system. It acts as an antioxidant to take care of free radicals, which can damage cells and DNA, which may help to the aging process. It also helps to reduce risk of developing cataracts, heart diseases, and cancer [28–30].

2-Methoxyestradiol is an endogenous metabolite of human hormone. It is used in patients with brain tumors, but it has significant adverse effects. It is commonly used in combination with quercetin to minimize the side effects [31]. The combination of quercetin and 2-methoxyestradiol looks very promising for treating patients with prostate cancer. Therefore, combination of quercetin and 2-methoxyestradiol can serve as a novel clinical treatment regimen owing to the potential of enhancing antitumor effect on prostate cancer in vivo and lessening the dose and side effects of either quercetin or 2-methoxyestradiol alone. These in vivo*,* results will lay a further solid basis for subsequent studies on this novel therapeutic regimen in human prostate cancer [31]. The physiological and biochemical functions of ascorbic acid, as electron donor, and can be used as an adjuvant in the treatment of various types of cancer [32]. Amiloride-HCL modulates oncogenic RNA Alternative Splicing to devitalize human cancer cells. Proteome complexity expanded by alternative splicing (AS), a process involving differential exon inclusion or exclusion of the same pre-mRNA molecule to produce various mRNA and protein isoforms [33–35]. Quinine sulfate is the natural product to treat malaria in humans [36].

Pancreatic cancer is the fourth-leading cause of death in the USA. Tocotrienols are better anti-oxidants than tocopherols due to its unsaturated side-chain, which facilitate better penetration into *trans* saturated fatty layers of liver and brain [37, 38]. Tocotrienols inhibit tumor formation, and very effective in reducing human pancreatic carcinoma cells and BxPC-3 pancreatic ductal adenocarcinoma cells [39]. Tocotrienols are found to be very effective in human breast cancer cells and for inducing apoptosis in estrogen-responsive and estrogen-nonresponsive human breast cancer cells by targeting cancer cells by inhibiting Id1, a key cancer-promoting protein [40]. This mechanism was also observed in prostate cancer and melanoma cell lines [41]. γ-Tocotrienol is very potent for cell apoptosis and anti-proliferation of cancer cells [40]. The anti-proliferative effect of tocotrienols reported in prostate cancer cells by detoxification mechanism. γ-Tocotrienol was potent in suppressing prostate cancer proliferation, this anti-proliferative effect is through multiple-signaling pathways (NF-κB, EGF-R, Id family proteins) [42]. Tocotrienols have also found to be effective against human malignant melanoma cells [43]. In short, all these published properties clearly indicate the importance of these compounds tested in vitro in cancer cell lines of different organs in various types of cancer. Future investigation may explore their effects alone or as combined therapy, specifically with naturally-occurring compounds in vivo to treat various types of cancer in humans, as it is well known that consumption of moderate doses of naturally-occurring compounds have no side-effects in humans.

We have shown that proteasome plays a pivotal role in modulating lipopolysaccharides (LPS)-induced inflammation [12] Proteasomes have several functions and degrade crucial regulatory proteins via its chymotrypsin-like (X, LMP7), post-acidic (Y, LMP2) and trypsin-like (Z, LMP10) activities. We have also shown that different mouse/human cells differ in the proteasome subunits they contain. Therefore, upregulation/downregulation proteasome’s activities play a major role in metabolism and cellular innate immune response [44]. These results suggest that expression of proteasome LMP subunits and TNF-α are clearly down regulated after δ-tocotrienol treatment, as described earlier [20–24] that down-regulation of proteasome subunits leads to apoptosis and autophagy of cells [45]. We have also noted that gene expression of all proteasome subunits significantly reduced in whole-blood cells obtained from hepatitis C patients, relative to healthy volunteers. Concurrently, protein expression levels of VCAM-1, ICAM-1, resistin, biomarkers in plasmas are clearly up regulated during initial stage of cancer, but significantly down regulated in later stages of cancer in patients.

## Conclusions

The present in vitro results indicate that δ-tocotrienol is very potent proteasome inhibitor among other outstanding inhibitors (such as thiostrepton, 2-methoxyestradiol, quercetin) for apoptosis tested in all nine cell lines (Hela, liver, pancreatic, prostrate, breast, melanoma, B lymphocytes, T-cells). The total RNAs obtained from plasmas of δ-tocotrienol treated hepatitis C patients showed significant decreases in the expression of TNF-α (47%) and VCAM-1 (22%) pro-inflammatory cytokines. However, there was a robust increase in the expression of ICAM-1 (96%) and IFN-γ (35%). Expression of the proteasome subunits X, Y, Z (10% - 22%) and LMP7, LMP2, LMP10 (24% - 44%) decreased significantly after δ-tocotrienol treatment. These results suggest that expression of proteasome subunits and TNF-α were down regulated after δ-tocotrienol treatment, and excessive down-regulation of proteasome subunits leads to autophagy and apoptosis of cells. These results are further supported by RNA-sequence analysis by Ingenuity Pathway Analysis (IPA) of plasma RNAs obtained from δ-tocotrienol treatment of hepatitis C patients, and will be the subject of our next manuscript. That will describe possible canonical pathways, up-stream regulators, diseases and functional metabolic networks using δ-tocotrienol. Collectively, these results highlight the critical importance for role of proteasomes in inflammation and apoptosis of cells. Furthermore, total RNAs purified from plasma of hepatitis C patients accurately reflects key changes in levels of proteasome subunits and will help to distinguish patients with normal, and possible inflammatory diseases. Moreover, addition of selected proteasome activators/inhibitors to cultures of these cells will enhance or suppress cellular responsiveness depending on the type of cell. Since, the proteasome affects so many pathways (ubiquitin-proteasome, Toll-like, STAT3) present data have revealed that some of these proteasome activators may be useful in several types of cancer patients and other inflammatory diseases.

## Abbreviations

ICAM1: Intercellular adhesion molecule1
IL-6: Interleukin-6
IPA: Ingenuity Pathway Analysis
NF-κB: Nuclear factor kappaB
PPAR-α: Peroxisome proliferator-activated receptor-α
TNF-α: Tumor necrosis factor-α
VCAM1: Vascular cell adhesion molecule1

## References

[1] Brown III CH, Baidas SM, Hajdenberg JJ, Kayaleh OR, Pennock GK, Shah NC, Tseng JE (2000). Lifestyle interventions in the prevention and treatment of cancer. Am J Lifestyle Med.

[2] Google Search (2015). Cancer: facts, causes, symptoms and research. Medical news today.

[3] Bhat UG, Zipfel PA, Tyler DS, Gartel AL (2008). Novel anticancer compounds induce apoptosis in melanoma cells. Cell Cycle.

[4] Bhat UG, Halasi M, Gartel AL (2009). Thiozole antibiotics target FoxM1 and induces apoptosis in human cancer cells. PLoS One.

[5] Bhat UG, Halasi M, Gartel AL (2009). FoxM1 is a general target for proteasome inhibitors. PLoS One.

[6] Pandit B, Bhat UG, Gartel A (2011). Proteasome inhibitory activity of thiozole antibiotics. Cancer Biol Ther.

[7] Gartel AL (2010). A new target for proteasome inhibitors: FoxM1. Expert Opin Investig Drugs.

[8] Pilarsky C, Wenzig M, Specht T, Saeger HD, Grutzmann R (2004). Identification and validation of commonly overexpressed genes in solid tumors by comparison of microarray data. Neoplasia.

[9] Wang M, Gartel AL (2011). Micelle-encapsulated thiostrepton as an effective nanomedicine for inhibiting tumor growth and for suppressing FOXM1 in human xenografts. Mol Cancer Ther.

[10] Lyass O, Uzeily B, Ben-Yosef R, Tzemach D, Heshing NI, Lotem M, Brufman G, Gabizon A (2000). Correlation of toxicity with pharmacokinetics of pegylated liposomal doxorubicin (Doxil) in metastatic breast carcinoma. Cancer.

[11] Qureshi AA, Tan X, Reis JC, Badr MZ, Papasian CJ, Morrison DC, Qureshi N (2011). Suppression of nitric oxide induction and pro-inflammatory cytokines by novel proteasomes inhibitors in various experimental models. Lipids Health Dis.

[12] Qureshi AA, Reis JC, Badr MZ, Silswal N, Qureshi N (2017). Selected compounds modulate various inflammatory biomarkers in lipopolysaccharide-induced macrophages of PPAR-α mice. J Clin Exp Res Cardiol.

[13] Hosseini A, Ghorbani A (2015). Resveratrol and clinical cancer studies. Avicenna J Phytomed.

[14] Hsieh TC, Wu JM. Resveratrol properties as anticancer molecule. Biofactors. 2010; 10.1002/biof.105. 10.1002/biof.105 PMC3655417 20623546

[15] Savouret JF, Quesne M (2002). Resveratrol and cancer: a review. Biomed Pharmacother.

[16] Qureshi AA, Mo H, Packer L, Peterson DM (2000). Isolation and structural identification of noveln tocotrienols from rice bran with hypocholesterolemic, antioxidant and antitumor properties. J Agr Food Chemistry.

[17] Shen J, Reis JC, Morrison DC, Papasian CJ, Sreekumar R, Kolbert C, Qureshi AA, Vogel SN, Qureshi N (2006). Key inflammatory signaling pathways are regulated by proteasomes. Shock.

[18] Qureshi N, Perera PY, Splitter G, Morrison DC, Vogel SN (2005). The proteasome as a LPS-binding protein in macrophages: toxic lipopolysaccharide activates the proteasome complex. J Immunol.

[19] Shen J, Gao JJ, Zhang G, Tan X, Morrison DC, Papasian CJ, Vogel SN, Qureshi N (2006). Proteasome inhibitor, lactacystin blocks CpG DNA- and peptidoglycan induced inflammatory genes, cytokines and mitogen-activated protein kinases in macrophages. Shock.

[20] Zheng Q, Wang Q, Wang S, Wu J, Gao Q, Liu W (2015). Thiopeptide antibiotics exhibit a dual mode of action against intracellular pathogens by affecting both host and microbe. Chem Biol.

[21] Ziparo E, Petrungaro S, Marini ES, Starace D, Conti S, Facchiano A, Filippini A, Giampietri C (2013). Autophagy in prostate cancer and androgen suppressioin therapy. Int J Mol Sci.

[22] Rubinsztein DC, Bento CF, Deretic V (2015). Therapeutic targeting of autophagy in neurodegenerative and infectious diseases. J Exp Med.

[23] Nedelsky NB, Todd PK, Taylor JP (2008). Autophagy and ubiquitin-proteasome system: collaborators in neuroprotection. Biochim Biophys Acta.

[24] Zhu K, Dunner K, McConkey DJ (2010). Proteasome inhibitors activate autophagy as a cytoprotective response in human prostate cancer cells. Oncogene.

[25] AHFS DRUG INFORMATION (2006). American society of health-system pharmacists.

[26] Brunton LL, Chabner BA, Knollman BC. Goodman and Gilman’s the pharmacological basis of therapeutics, 12th ed. Chapter 53. New York: Mcgraw-Hill; 2011. p. 463-76.

[27] Davies S, Dai D, Pickett G, Leslie KK (2006). Gene regulation profiles by progesterone and dexamethasone in human endometrial cancer Ishikawa H cells. Gynecology. Oncology.

[28] Colombo B, Saraceno L, Comi G (2014). Riboflavin and migraine: the bridge over troubled mitochondria. Neurol Sci.

[29] Cumming RG, Christian P, Smith W (2000). Diet and cataract: the Blue Mountains eye study. Ophthalmology.

[30] Fishman SM, Christian P, West KP (2000). The role of vitamins in the prevention and control of anaemia. Pulic Health Nutr.

[31] Yang F, Song L, Wang H, Wang J, Xu Z, Xing N (2015). Combination of quercetin and 2-methoxyestradiol enhances inhibition of human prostate cancer LNCaP and PC-3 cells xenograft tumor growth. PLoS One.

[32] Du J, Cullen JJ, Buettner GR (2012). Ascorbic acid: chemistry, biology and treatment of cancer. Biochim Biophys Acta.

[33] Chang JG, Yang DM, Chang WH, Chow LP, Chan WL, Lin HH, Huang HD, Chang YS, Hung CH, Yang WK (2011). Small molecule amiloride modulates oncogenic RNA alternative splicing to devitalize human cancer cells. PLoS One.

[34] Maniatis T, Tasic B (2002). Alternative pre-mRNA splicing and proteasome expansion in metazoans. Nature.

[35] Stetefeld J, Rugg MA (2005). Structural and functional diversity generated by alternative in mRNA splicing. Trends Biochem Sci.

[36] Krishna S, White NJ (1996). Pharmacokinetic of quinine, chloroquinine: clinical implications. Clin Pharmacokinet.

[37] Kamat JP, Sarma HD, Devasagayam TP, Nesaretnam K, Basiron Y (1997). Tocotrienols from palm oil as effective inhibitors of protein oxidation and lipid peroxidation in rat liver microsomes. Mol Cell Biochem.

[38] Kamat JP, Devasagayam TP (1995). Tocotrienols from palm oil as potent inhibitors of lipid peroxidation and protein oxidation in rat brain mitochondria. Neurosci Lett.

[39] Hussein D, Mo H (2009). D-*delta*-Tocotrienol-mediated suppression of the proliferation of human PANC-1, MIA PaCa-2, and BxPC-3 pancreatic carcinoma cells. Pancreas.

[40] Nesarenam K, Ambra R, Selvaduray KR, Radhakrishnan A, Canali R, Virgili F (2004). Tocotrienol rich fraction from palm oil and gene expression in human breast cancer cells. Ann N Y Acad Sci.

[41] Yap WN, Zaiden N, Tan YL, Ngoh CP, Zhang XW, Wong YC, Lin MT, Yap YL (2009). Cancer Lett.

[42] Yap WN, Chang PN, Han HY, Lee DT, Ling MT, Wong WC, Yap YL (2009). γ-Tocotrienol suppress prostate cancer cell proliferation and invasion through multiple signaling pathways. Br J Cancer.

[43] Chang PN, Yap WN, Lee DT, Ling MT, Wong YC, Yap YL (2009). Evidence of gamma-tocotrienol as an apoptosis-inducing, invasion-suppressing, and chemotherapy drug-sensitizing agent in human melanoma cells. Nutr Cancer.

[44] Reis JC, Tan X, Yang R, Qureshi AA, Papasian CJ, Vogel SN, Morrison DC, Qureshi NA (2008). Combination of proteasome inhibitors and antibiotics prevent lethality in septic shock model. Innate Immun.

[45] Korolchuk VI, Menxies FM, Rubinsztein DC (2010). Mechanism of cross-talk between the ubiquitin-proteasome and autophagy-lysosome systems. FEBS Lett.

